# The Effects of Herbivory by a Mega- and Mesoherbivore on Tree Recruitment in Sand Forest, South Africa

**DOI:** 10.1371/journal.pone.0017983

**Published:** 2011-03-22

**Authors:** D. D. Georgette Lagendijk, Robin L. Mackey, Bruce R. Page, Rob Slotow

**Affiliations:** Amarula Elephant Research Programme, School of Biological and Conservation Sciences, University of KwaZulu-Natal, Durban, South Africa; Duke University, United States of America

## Abstract

Herbivory by megaherbivores on woody vegetation in general is well documented; however studies focusing on the individual browsing effects of both mega- and mesoherbivore species on recruitment are scarce. We determined these effects for elephant *Loxodonta africana* and nyala *Tragelaphus angasii* in the critically endangered Sand Forest, which is restricted to east southern Africa, and is conserved mainly in small reserves with high herbivore densities. Replicated experimental treatments (400 m^2^) in a single forest patch were used to exclude elephant, or both elephant and nyala. In each treatment, all woody individuals were identified to species and number of stems, diameter and height were recorded. Results of changes after two years are presented. Individual tree and stem densities had increased in absence of nyala and elephant. Seedling recruitment (based on height and diameter) was inhibited by nyala, and by elephant and nyala in combination, thereby preventing recruitment into the sapling stage. Neither nyala or elephant significantly reduced sapling densities. Excluding both elephant and nyala in combination enhanced recruitment of woody species, as seedling densities increased, indicating that forest regeneration is impacted by both mega- and mesoherbivores. The Sand Forest tree community approached an inverse J-shaped curve, with the highest abundance in the smaller size classes. However, the larger characteristic tree species in particular, such as *Newtonia hildebrandtii*, were missing cohorts in the middle size classes. When setting management goals to conserve habitats of key importance, conservation management plans need to consider the total herbivore assemblage present and the resulting browsing effects on vegetation. Especially in Africa, where the broadest suite of megaherbivores still persists, and which is currently dealing with the ‘elephant problem’, the individual effects of different herbivore species on recruitment and dynamics of forests and woodlands are important issues which need conclusive answers.

## Introduction

Different-size herbivores have different feeding preferences [1]; however, some overlap, and hence competition, might exist between different trophic guilds [2]. Megaherbivores (body mass ≥1000 kg [3]) compete with mesoherbivores (medium-size herbivores with body mass between 50 and 450 kg [1], [2]) for food [2] as they feed in overlapping height ranges [4], [5]. Through their browsing activities, both mega- and mesoherbivores have the capacity to alter structural diversity (e.g. height class distributions) of forests and woodlands [6], [7]. Some megaherbivores open up the canopy by changing the vertical structure from top down, by impacting on large trees and browsing at higher levels [3]. On the other hand, mesoherbivores may have considerable effects as (1) controllers of the state induced by megaherbivores, by suppressing woodland or forest recovery through browsing after megaherbivore impact has altered woodland to shrubland [8] or (2) top down control of recruitment into taller height classes by browsing of seedlings [9], [10]. Individual species or entire communities may disappear over time when there is no adequate recruitment and hence regeneration into taller height classes to compensate natural die-offs, impact of fire [11] and megaherbivores.

While numerous studies have focused on megaherbivore impact on woody communities (e.g. [3], [12]–[16]), and on the effects of herbivores in general on community structure and composition [17]–[21], the combined effects of both mega- and mesoherbivore species on different height classes have received scant attention. Exclosure experiments in savanna landscapes have tried to separate effects on vegetation by different groups of herbivores [22]–[26]. However, effects observed in these studies can not be positively ascribed to one species only, when distinguishing between groups of herbivores of similar sizes. Consequently, the specific browsing effects of both mega- and mesoherbivores on regeneration of woody vegetation, especially in the African context where the broadest suite of megaherbivores still persists, still remain largely unknown.

Here we focus on the impacts on seedling and sapling recruitment by a mega- and mesoherbivore within the critically endangered Sand Forest community [27]. This deciduous dry forest type is restricted to the Maputaland Centre of Endemism in north-eastern KwaZulu-Natal, South Africa and southern Mozambique [28]–[30]. Sand Forest generally occurs in a mosaic of patches enclosed by mixed woodland or savanna bushveld [31], [32], and includes a large number of rare and endemic species [29], [30], [32]. Its restricted geographic range and unique species composition makes Sand Forest one of the most important habitat types for conservation in southern Africa [28], [31], [33]. Sand Forest is susceptible to fire and selective species utilisation by both man and herbivores, the effects of which are exacerbated by Sand Forest's low resilience to disturbance and poor recruitment rates of its tree species [29], [34], [35]. While foraging, browsing herbivores create pathways which open up the forest [36], [37]. Once savanna vegetation enters these gaps within the Sand Forest, successive changes to savanna woodland may be irreversible [31], [34].

The dynamics of Sand Forest are poorly understood [32]. The structural diversity in the Sand Forest system in some protected areas has changed drastically over the past decade, particularly in Tembe Elephant Park (Matthews pers. comm.) and Phinda Private Game Reserve (Pretorius pers. comm.). The main reason for this is thought to be herbivory [5], [38], affecting both the recruitment phase and taller height classes. Both elephant *Loxodonta africana* and nyala *Tragelaphus angasii* became locally abundant in protected Sand Forest areas after (re)introductions of these species in the early 1990s.

Conservation of the Sand Forest community is of critical importance, and it is therefore imperative to assess potential drivers affecting the tree community and its low recruitment rates. Management questions have been raised regarding the impact of herbivores, in particular elephants [39], on the vegetation, such as whether elephants or other herbivores are causing irreversible damage to the Sand Forest ecosystem, and if densities of these species need to be reduced in order to conserve the forest. We hypothesise that both mixed feeders have had, and are having, substantial impact on the vegetation [5], [29], [38], as densities of both elephant and nyala have increased since (re)introduction with concomitant changes to Sand Forest structural diversity. Therefore, the aim of this study was to assess the role of elephant and nyala on Sand Forest structure through their individual and combined browsing effect, particularly on recruitment. While impala *Aepyceros melampus* have been linked to recruitment limitation [10], [40], this has not been studied for nyala. To our knowledge, this is the first study to experimentally separate the browsing effects of a mega- and mesoherbivore.

## Methods

### Study area

Phinda Private Game Reserve (hereafter Phinda) is a 180 km^2^ (27°92′–27°68′S; 32°44′–32°20′E) conservation area in Maputaland, northern KwaZulu-Natal, South Africa. The reserve includes a wide range of habitat types, such as western Maputaland sandy bushveld as well as several patches of the endemic Sand Forest [27]. The climate is subtropical with hot, humid summers and warm, dry winters. Temperatures range from a minimum of 10°C in winter to a maximum of 35°C in summer. Annual rainfall ranges between 350 mm and 1100 mm, and varies spatially from west to east.

Before Phinda was created in 1991, the area consisted of private and small game farms. Game was introduced following the establishment of the park [38], with fifty-eight elephants being released into Phinda between 1992 and 1994 [41]. At the start of this study (2005) 81 elephants were present in the reserve, increasing to 98 individuals in 2007 (based on an individually identified and monitored elephant population (e.g. [42])). Nyala numbered approximately 1100 and 1750 individuals in 2005 and 2007, respectively (based on annual aerial game counts). Other browsing ungulates in Phinda include giraffe *Giraffa camelopardalis* (154), kudu *Tragelaphus strepsiceros* (188), impala (1690), red duiker *Cephalophus natalensis* (23), common duiker *Sylvicapra grimmia* (no count available) and suni *Neotragus moschatus* (no count available). Counts in parentheses are approximate and reflect the 2007 annual helicopter game count.

This study was conducted in the endemic Sand Forest, which occurs in the northern section of Phinda. Sand Forests occur on acidic, sandy soils with very little clay [29]. The Sand Forest is a dense vegetation type, with a closed canopy, 5 to 12 m high, without a significant understorey. Characteristic woody species include *Balanites maughamii*, *Cleistanthus schlechteri*, *Cola greenwayi*, *Croton pseudopulchellus*, *Dialium schlechteri*, *Drypetes arguta*, *Hymenocardia ulmoides*, *Newtonia hildebrandtii* and *Pteleopsis myrtifolia* [28], [31], [33]. Few mammal species utilise Sand Forest [31]. In Phinda, elephant and nyala are the *only* mega- and mesoherbivore utilising the Sand Forest patches (Lagendijk pers. obs.).

### Experimental design

**Figure 1:**
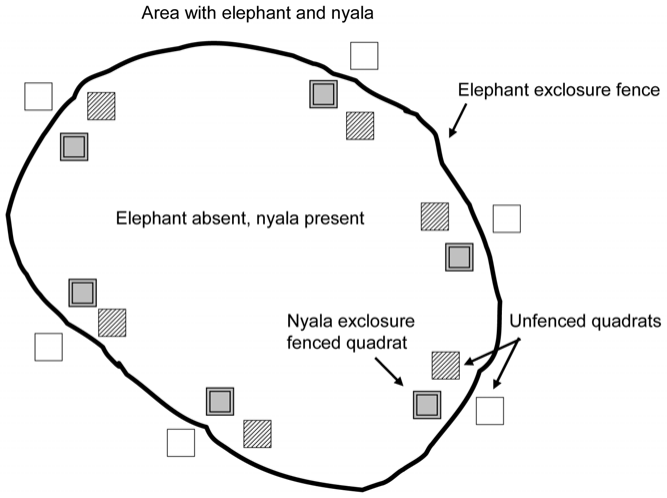
A schematic representation of the exclosure experiment with the three treatments. (1) open access, accessible for all herbivores (+E+N, open bars); (2) partial exclosure, elephant excluded, nyala present (−E+N, diagonal hatching); (3) full exclosure, both elephant and nyala excluded (−E−N, grey bars) (not to scale). The sets of three treatments were replicated 12 times.

The effects of elephant and nyala on Sand Forest recruitment were tested using exclosures. In November 2005, elephants were excluded from part of the Sand Forest using electrified (7000 volts per second) high tension galvanized wires (2.4 mm thick) erected at 1.8 m and 2 m above the ground, enclosing 3.09 km^2^ of the 5.2 km^2^ Sand Forest patch (Fig. 1). To determine the effects of both elephant and nyala separately, twelve exclosures of 20 m×20 m using 1.8 m high bonnox fencing (a coarse wire mesh with 30×20 cm openings) were erected inside the elephant-free area. This type of fencing allowed passage for small-size herbivores such as duiker and suni, but excluded nyala. Adjacent to this exclosure, another 20 m×20 m area was marked out and opposite these two treatments just outside the elephant fence a third 20 m×20 m area was marked for sampling. This resulted in an experimental design of a set of three 400-m^2^ treatments in close proximity, consisting of: (1) unfenced area available to all herbivores (open access “+E+N”); (2) area fenced to exclude only elephant (partial exclosure, nyala present “−E+N”); (3) area fenced to exclude both nyala and elephants (full exclosure “−E−N”), but providing access to smaller herbivores. There were a total of 12 replicates of this set of three treatments. Distance between replicates ranged between 0.12 km and 2.75 km.

A base-line study was conducted in 2005. In each quadrat (n = 36) all woody individuals (including seedlings; >0.02 m and ≤0.5 m tall; and saplings, >0.05 m and ≤1.5 m tall) were identified to species and counted. Diameters above the buttress swelling of all stems (including seedlings and saplings) and all tree heights were recorded. Diameters and the heights of trees to 2 m were measured, the heights of trees between 2 m and 4 m estimated to the nearest 20 cm, and the heights of taller trees estimated to the nearest 50 cm using the height of an observer as a scale following Shannon et al. [16]. Two years after implementation of the experiment, quadrats were sampled again during June–July or November–December 2007, with the three treatments from a replicate being sampled during the same sampling trip. Five open access plots were repositioned in 2007, therefore when doing pair-wise comparisons between the two sampling years, only 7 open access plots (+E+N) were included in the analyses. The analyses are of 2007 data unless otherwise stated.

### Statistical analyses

Following recommendations by Clarke and Warwick [43], tree species contributing less than 4 percent of the abundance per plot in 2005 were discarded and only species present in both sampling years were included. Given that recruitment is dependent on a local seed source, we believe that excluding the rare species provides a more robust test of recruitment patterns across treatments. Consequently a total of 26 tree species were included in the analyses (Table 1); all of these species were browsed upon by the herbivore guild during the course of the experiment.

**Table 1:** List of 26 species included in the analyses.

*Cola greenwayi* Brenan	*Hymenocardia ulmoides* Oliv.	*Rhus natalensis* Bernh. Ex Krauss
*Combretum celastroides* Welw. Ex Laws.	*Hyperacanthus amoenus* (Sims) Bridson	*Salacia leptoclada* Tul.
*Combretum mkuzense* Carr & Retief	*Landolphia kirkii* T.-Dyer	*Strychnos henningsii* Gilg
*Croton pseudopulchellus* Pax	*Monanthotaxis caffra* (Sond.) Verdc.	*Toddalopsis bremekampi* Verdoorn
*Croton steemkampianus* Gerstner	*Monodora junodii* Engl. & Diels	*Tricalysia junodi* (Schinz) Brenan
*Dialium schlechteri* Harms	*Newtonia hildebrandtii* (Vatke) Torre	*Uvaria caffra* E. Mey. Ex Sond.
*Drypetes arguta* (Muell. Arg.) Hutch	*Ptaeroxylon obliquum* (Thunb.) Radlk.	*Wrightia natalensis* Stapf
*Grewia microthyrsa* K. Schum. Ex Burret	*Pteleopsis myrtifolia* (Laws.) Engl. & Diels	*Zanthoxylum* sp.
*Haplocoelum gallense* (Engl.) Radlk.	*Rhus gueinzii* Sond.	

Because there are two possible demographic responses to browsing viz. mortality or a coppicing response (i.e. the production of new stems after the terminal part of the main stem has been removed [44]), changes in both the density of individual trees (which measures mortality, but also reflects recruitment) and stem density (which measures the coppicing response or mortality of vertical stems) were investigated. Individual and stem densities were scaled up from 400 m^2^ to 1 ha. Individual trees and stems were allocated to seven height classes (≤0.5 m, 0.51–1.5 m, 1.51–3 m, 3.01–5 m, 5.01–8 m, 8.01–12 m, >12 m), which roughly correspond to the limits at which browsing by different-size herbivores occurs.

ANOVAs were used to test for differences in overall tree and stem densities among treatments in 2007, for which data were pooled for all species and height classes. Differences in seedling (≥0.02 and ≤0.5 m in height) and sapling (>0.5 m and ≤1.5 m in height) abundance, as well as stem densities in these height classes, among treatments were also analysed using ANOVA. Pair-wise comparisons of individual overall tree densities and seedlings per treatment between 2005 and 2007 were also analysed using ANOVA.

Tree populations are regenerating when the population structure displays an inverse J-shaped frequency distribution [45], [46]. This translates to a relatively high abundance of seedlings, which represents sufficient recruitment, and a relatively low abundance of tall trees. A distribution of a different shape is indicative of disturbance [47]. Following previous work in Sand Forest [48], [49], we used 18 different size classes with 1 cm intervals to 7 cm diameter, thereafter 2 cm intervals to 15 cm diameter, 5 cm intervals to 30 cm diameter and 10 cm intervals to 60 cm. The diameter limits that are equivalent to the height categories we used are 1, 4, 9, 15, 25, 40 and >40 cm diameter (derived from a quadratic regression of diameter vs height for all Sand Forest species (r^2^ = 0.73)). A G-test was used to determine whether size distributions differed among treatments for the pooled data. To prevent compounding of Type 1 errors from running three pair-wise G-tests, alpha of 0.05 was Bonferroni-adjusted to 0.017.

At the tree species level, we focused our analyses on the three most common Sand Forest species in our study area (*Salacia leptoclada*, *Uvaria caffra* and *Tricalysia junodii*) and on three characteristic Sand Forest trees (*D. schlechteri*, *N. hildebrandtii* and *P. myrtifolia*) to determine the effect of elephant and/or nyala on recruitment. Seedling and sapling abundance were analysed separately among treatments per focus species using a two-way ANOVA. When ANOVA assumptions were not met, densities were analysed with a Kruskal-Wallis test. Size class distributions (SCD), which reflect population structures, were analysed for each of these six species using linear regressions (cf. [45], [50]). Data were pooled per treatment. The number of individual trees per diameter class was divided by the width of the diameter class, giving an average density (D_i_) for the class midpoint (M_i_). These variables were ‘ln+1’ – transformed prior to regression analyses. All size classes up to the largest size class containing individuals were included in the analyses. We used SCD slopes to interpret population structures. An inverse J-shaped curve is represented by a steep negative slope, while species with little regeneration show a negative slope close to zero.

For all the abovementioned statistical tests the significance level was set at P = 0.05, unless otherwise stated. All significant ANOVAs (assumptions of normality and homoscedasticity being met) were followed-up with Tukey's post-hoc tests. All statistical analyses were performed using SPSS 15.0 (SPPS Inc., Chicago, USA).

## Results

In 2005, 12638 individual plants from 95 woody species, and in 2007, 17825 individual trees and 143 woody species were recorded in all treatments. In 2007, the dominant Sand Forest species *S. leptoclada*, *T. junodii*, and *U. caffra* made up 49.9% of all trees, compared to 53.4% in 2005.

The twelve replicates of the experiment were considered to be homogeneous in 2005 as, when only taking the more abundant species (n = 26) into account, there were no significant differences among treatments for the seedling (F_0.05(2)2,33_ = 0.363, P = 0.698), sapling (F_0.05(2)2,33_ = 0.944, P = 0.399), and overall (i.e. all size classes combined) tree and stem densities (F_0.05(2)2,33_ = 0.842, P = 0.440 and F_0.05(2)2,33_ = 1.905, P = 0.165 respectively). Note that when all species present were included in this analysis, there were still no significant differences for any of these contrasts (seedling: F_0.05(2)2,33_ = 1.044, P = 0.363; sapling: F_0.05(2)2,33_ = 0.758, P = 0.464; tree density for all size classes combined: F_0.05(2)2,33_ = 0.883, P = 0.444; stem density for all size classes combined: F_0.05(2)2,33_ = 2.734, P = 0.080).

In contrast to this, for the 26 species in 2007 there were significant differences among the treatments in both the overall tree densities (F_0.05(2)2,33_ = 5.180, P = 0.011) and the overall stem densities (F_0.05(2)2,33_ = 4.426, P = 0.020), with densities in the full exclosure (−E−N) being significantly greater than in the open access treatment (+E+N) (overall tree density, Tukey: P = 0.010; overall stem density, Tukey: P = 0.027). The overall abundance of individual trees in the partial exclosure (−E+N), were not significantly different from those in the open access (+E+N) or in the full exclosure (−E−N) treatment. However stem densities were greater in the full exclosure than in the partial exclosure, although this was marginally not significant (Tukey: P = 0.056). Pair-wise comparisons between 2005 and 2007 showed a significant increase in the full exclosure for overall tree densities (−E−N: F_0.05(2)1,22_ = 7.387, P = 0.013). Differences in overall stem densities per treatment between 2005 and 2007 were not significant (open access (+E+N): F_0.05(2)1,12_ = 0.599, P = 0.454; partial exclosure (−E+N): F_0.05(2)1,22_ = 0.537, P = 0.471; full exclosure (−E−N): F_0.05(2)1,22_ = 2.401, P = 0.136). This indicated that recruitment was taking place within the full exclosure (−E−N).

**Figure 2:**
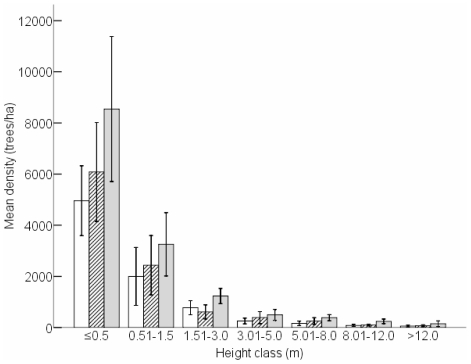
Mean density (trees/ha) per height class (i.e. seedlings: ≤0.5 m; saplings; 0.51–1.5 m) for all 26 species combined per treatment. Open access (+E+N, open bars), partial exclosure (−E+N, diagonal hatching) and full exclosure (−E−N, grey bars). The bars indicate 95% confidence intervals of the means. N = 12 replicates per treatment.

**Figure 3:**
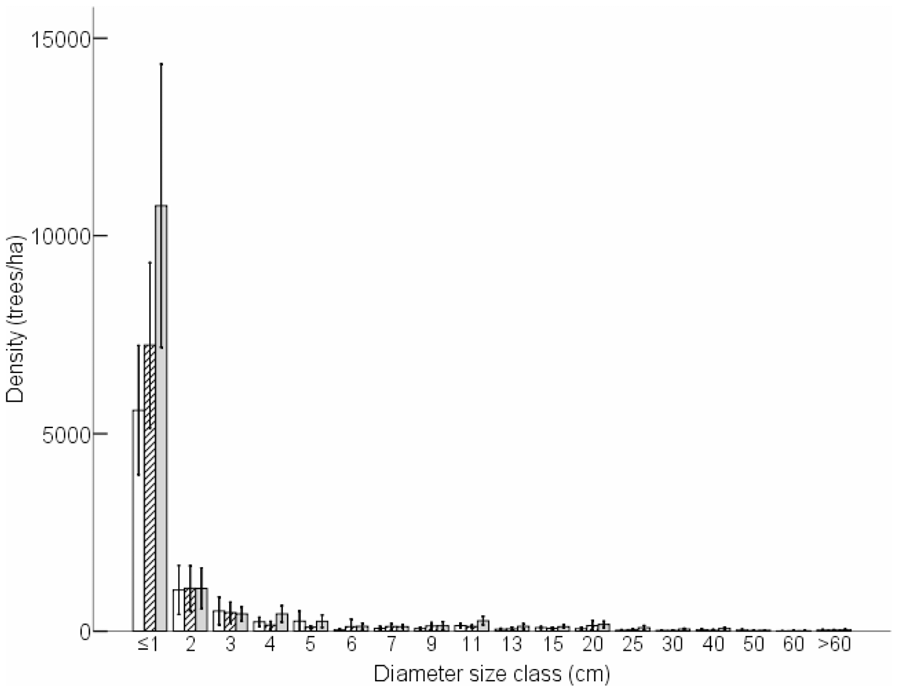
Mean density (trees/ha) per diameter size class (i.e. seedlings: ≤1 cm; saplings; 1–4 cm) for all 26 species combined per treatment. Open access (+E+N, open bars), partial exclosure (−E+N, diagonal hatching) and full exclosure (−E−N, grey bars). The bars indicate 95% confidence intervals of the means. N = 12 replicates per treatment.

Seedling density of the 26 species differed significantly among treatments in 2007 (F_0.05(2)2,33_ = 3.582, P = 0.039; Fig. 2). Seedling densities in the full exclosure (−E−N) were significantly higher than in the open access (+E+N) treatment (Tukey: P = 0.035), indicating that both nyala and elephant in combination reduced seedling densities. This is concordant with analysing seedling densities by tree diameter class as opposed to height class. Seedling density (0–1 cm diameter class) differed significantly among treatments (F_0.05(2)2,33_ = 5.104, P = 0.012), with greater seedling densities in the full exclosure (−E−N) than in the open access treatment (+E+N: Tukey: P = 0.010; Fig. 3).

**Figure 4:**
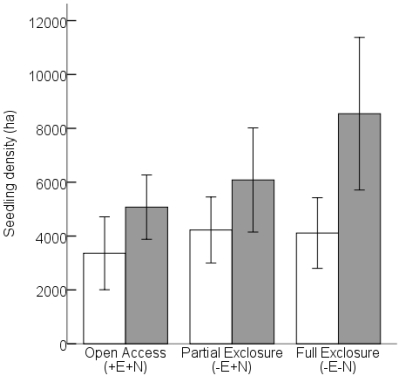
Mean seedling density (trees/ha) for all 26 species combined per treatment per sampling year. Open bars: 2005; grey bars: 2007. The bars indicate 95% confidence intervals of the means. N = 7 replicates for the open access treatment (+E+N) and N = 12 for the partial (−E+N) and full exclosure (−E−N).

For the 26 species, pair-wise comparisons of seedling (≤0.5 m in height) density between 2005 and 2007 was not significantly different within the partial exclosure (−E+N: F_0.05(2)1,22_ = 3.186, P = 0.088). However, there was a significant increase in seedling densities in the open access treatment (+E+N: F_0.05(2)1, 12_ = 5.386, P = 0.039) and the full exclosure between 2005 and 2007 (−E−N: F_0.05(2)1,22_ = 9.755, P = 0.005; Fig. 4).

For the 26 species, sapling densities in 2007 were not significantly different among treatments (F_0.05(2)2,33_ = 1.421, P = 0.256; Fig. 2), and there were no significant differences in sapling densities within each treatment between the two sampling years (P>0.21). Using stem diameter as opposed to height, there were also no significant differences in densities of saplings (1.01 to 4 cm diameter class) among treatments in 2007 (F_0.05(2)2,33_ = 0.123, P = 0.884; Fig. 3).

**Figure 5:**
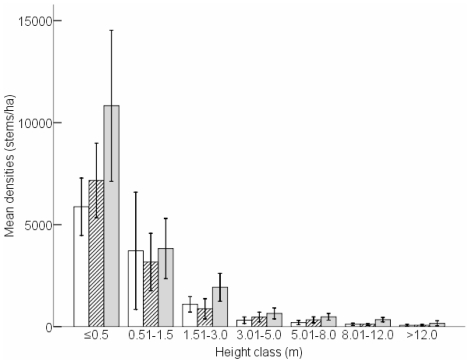
Mean stem density (stems/ha) per height class (i.e. seedlings: ≤0.5 m; saplings; 0.51–1.5 m) for all 26 species combined per treatment. Open access (+E+N, open bars), partial exclosure (−E+N, diagonal hatching) and full exclosure (−E−N, grey bars). The bars indicate 95% confidence intervals of the means. N = 12 replicates per treatment.

Seedling stem densities in 2007 were significantly different among the three treatments (F_0.05(2)2,33_ = 5.030, P = 0.012; Fig. 5), with seedling stem densities significantly greater in the full exclosure (−E−N) (Tukey: P = 0.012) than the open access treatment (+E+N). Sapling stem densities were not significantly different among treatments (F_0.05(2)2,33_ = 0.146, P = 0.865; Fig. 5). The greater seedling stem densities in the full exclosure also indicate that the differences in the density of individual trees are mostly due to recruitment of individual trees. However, additional stems were added from the recruitment of multi-stemmed trees or from the production of new stems from coppicing as a response to browsing prior to the establishment of the experiment.

Population structures were assessed using diameter size distributions for all species combined. In 2007, diameter size distributions were significantly different among treatments (G_12_ = 3169, P≤0.017 for all pair-wise comparisons). In all treatments, the highest abundance was found in the smallest size (≤1.0 cm) class (Fig. 3). Population structures approached an inverse J-shaped curve.

**Figure 6:**
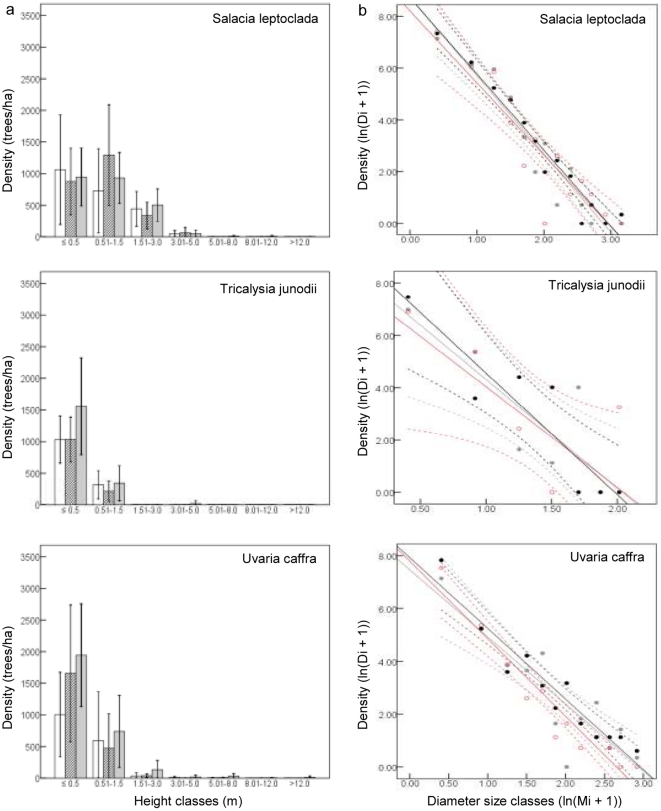
Size distribution curves of three common Sand Forest species in the three treatments. a, height class distribution (i.e. seedlings: ≤0.5 m; saplings; 0.51–1.5 m); b, linear regression of diameter class distribution. Open access (+E+N, open bars, grey circles and lines), partial exclosure (−E+N, diagonal hatching, red circles and lines) and full exclosure (−E−N, grey bars, black circles and lines). The bars (a) and dotted lines (b) indicate 95% confidence intervals. N = 12 replicates per treatment.

**Figure 7:**
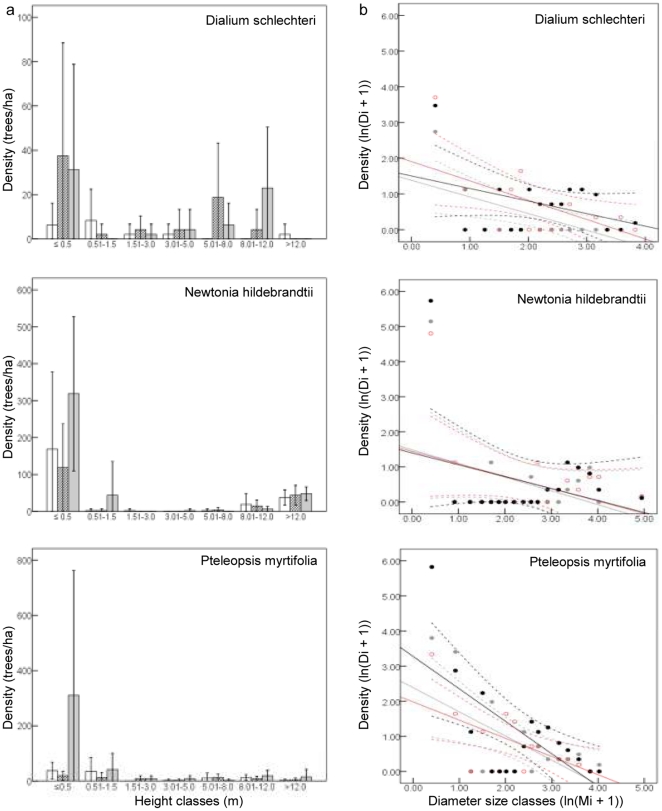
Size distribution curves of three characteristic Sand Forest species in the three treatments. a, height class distribution (i.e. seedlings: ≤0.5 m; saplings; 0.51–1.5 m); b, linear regression of diameter class distribution. Open access (+E+N, open bars, grey circles and lines), partial exclosure (−E+N, diagonal hatching, red circles and lines) and full exclosure (−E−N, grey bars, black circles and lines). The bars (a) and dotted lines (b) indicate 95% confidence intervals. N = 12 replicates per treatment.

Both seedling and sapling densities of each of the six selected focus species did not significantly differ among treatments (seedlings: P>0.54; saplings: P>0.33; Fig. 6a, 7a). However, the population structures of each of these six species had missing diameter size classes (mainly middle size classes). The population structures of *S. leptoclada*, *U. caffra* and *T. junodii* approached the inverse J-shaped curve characteristic of increasing populations, which is supported by the strong negative SCD slopes for these species (Fig. 6b, 7b, Table S1). *D. schlechteri*, *N. hildebrandtii* and *P. myrtifolia* showed a SCD slope closer to zero, indicating a disruptive population structure with little regeneration. However this was not significant for *N. hildebrandtii* (in any of the treatments) and *D. schlechteri* (in the full exclosure (−E−N)).

## Discussion

In addition to any effect small herbivores, rodents and invertebrates may have on recruitment [7], [22], [23], we show that forest regeneration is also impacted by both mega- and mesoherbivores as we managed to experimentally separate the browsing effects of elephant and nyala on recruitment. Both elephant and nyala potentially forage on recruiting individuals as the preferred feeding height of elephant falls between 1.0 and 2.0 m [4], and that of nyala between 0.6 and 1.1 m [5].

Neither seedlings nor saplings of the three common and three characteristic focus species showed a significant effect from browsing. Elephants have been found to select for *D. schlechteri*, *N. hildebrandtii*, *P. myrtifolia* and *T. junodii*, and use *S. leptoclada* less selectively in Sand Forest in Tembe Elephant Park (TEP) (*U. caffra* does not occur in TEP) [31]. However, it may well be that elephant in Phinda do prefer the first four species, but do not impact on the seedlings or saplings. To our knowledge, feeding preferences of nyala have not yet been published. In addition, Sand Forest soil seed banks have been found to be poor in TEP [32], which is consistent with the low seed bank densities for dry tropical forests [32]. Together with the short time frame of this study, this might explain the absence of a browsing effect on our focus species.

All three large tree species (*D. schlechteri*, *N. hildebrandtii* and *P. myrtifolia*) had size classes missing in the middle size cohorts, which may be explained by previous human utilisation of stems. In the last 25 years, the human population in the region [33], [51] has drastically increased with a concurrent intensification of the use of forest products, such as construction timber, fuel wood, wood for curios and medicinal plants [52], [53]. Missing size classes may also be a result of poor recruitment in the past; however there is little consensus over the potential causes underlying low recruitment rates in Sand Forest, which range from climatic factors (e.g. drought), periodic recruitment events, to browsing pressure [31], [32], [54].

Regeneration success, and hence recruitment of woody species into taller height classes, is dependent on a variety of factors. Seedling mortality is size-dependent, with the highest mortality occurring in the height class below 10 cm [55]. This implies that seedlings are most at risk during the establishment phase, when young trees are often most palatable [56]. Therefore, seedlings may need to escape a “browsing trap” (held in a height class making them more vulnerable to browsers) [57] induced by small- and medium-size herbivores [9], [10], [58], before being able to grow into the sapling phase. Our results support this as we found increased survival of individual trees and stems where both nyala and elephant were excluded, suggesting that browsing pressure may have been a limiting factor for Sand Forest recruitment in the past. This is strengthened by the relatively higher stem density in the full exclosure (−E−N). Trees within the seedling height which were browsed just prior the initiation of the experiment may have coppiced by 2007 after browsing release. This indicates a continued browsing pressure in the other treatments, and an inhibition of recruitment due to browsing. In addition, seedling densities had increased within the open access treatment (+E+N) and the full exclosure (−E−N) between the sampling years, but not in the partial exclosure (−E+N). This could be due to spatial heterogeneity in seed rain between treatments, but is more likely to be caused by increased browsing by nyala in absence of elephant [59] suppressing recruitment in the partial exclosure (−E+N). This effect of nyala is supported by the higher seedling densities found in the full exclosure (−E−N) from which they are excluded, than in the open access treatment (+E+N) where they are present with elephant.

While our research was conducted in one single Sand Forest patch, and we should thus be cautious with the interpretation of our results, we do believe that the mechanisms described here are applicable to other Sand Forest patches and other forest systems. Woodland populations are believed to benefit from a release from browsing pressure by megaherbivores [23], [36], [60]. Our findings (cf. [10], [40]) argue this viewpoint as we show that also the effects of mesoherbivores in combination with megaherbivores on forests dynamics cannot be ignored. This illustrates that while attention is often focussed on the individual herbivore species, the importance of browsing effects by multiple species on vegetation has often been neglected. Therefore effects of both mega- and mesoherbivores need to be taken into account when conserving woodlands and forests. This is especially important in the context of the ‘elephant problem’ [39], where conservation managers are concerned with the impacts of increasing elephant population densities on the environment, which may lead to the loss of tall trees and possibly to the conversion of woodland to grassland [36], [61]. While elephants can alter the vertical structure of vegetation from top down by impacting on tall trees, we show that both mega-and mesoherbivores in combination and nyala on their own, also have a strong top down effect on seedlings in forests (cf. [40] as a comparison to riparian woodlands for impala only), thereby preventing recruitment into taller height classes.

While two years of exclusion from browsers is a short time scale to observe changes in overall tree population structures (e.g. of individual species or in the larger height classes), this experiment shows that by manipulating disturbance factors (e.g. herbivory), changes in recruitment can be demonstrated within a short time interval (cf. [7]; 3 years). The exclosures as presented in this study are being maintained for long-term monitoring to better understand the effects of herbivores on woody vegetation. Our results suggest that the traditional notion that recruitment of Sand Forest is uncommon [31], [54] might be a misconception. We show that recruitment is taking place, at least into the seedling phase, but that further recruitment into taller height classes is prevented by strong browsing pressure. Certainly, the importance of browsing, and especially of multiple browsers, needs to be carefully considered in management planning for conservation areas.

We emphasise here the need to consider all possible factors influencing tree communities, and not only the “obvious” or “political” ones. In the case of Sand Forest, while fencing elephants from the Sand Forest will provide a reduction in damage to larger trees [16], [62], it would be critical to also exclude mesoherbivores in order to promote seedling recruitment and thus long-term sustainability of the few remaining Sand Forest patches in Southern Africa.

Since tourism revenues are an important source of income for most parks, the creation of botanical reserves within the protected area can be a lucrative management strategy. This type of management approach could also be applicable to other natural systems.

## Supporting Information

Table S1 **Regression analyses for tree abundance vs. tree diameter.**
(DOC)
