# Supplementary material for: The Effects of Herbivory by a Mega- and Mesoherbivore on Tree Recruitment in Sand Forest, South Africa
Source: PLoS One. 2011 Mar 22;6(3):e17983. doi: 10.1371/journal.pone.0017983 (PMC3062551; doi:10.1371/journal.pone.0017983)
Supplement: Table S1 — Regression analyses for tree abundance vs. tree diameter. (DOC) [file pone.0017983.s001.doc]

**Table 2. Regression analyses for tree abundance vs. tree diameter.**

| Species | Treatment | Slope | Intercept | R^2^ | Significance |
| --- | --- | --- | --- | --- | --- |
| *Salacia leptoclada* | Open access (+E+N)^a^ | -3.024 | 8.730 | 0.917 | ****** |
|  | Partial exclosure (-E+N)^b^ | -2.776 | 8.204 | 0.842 | ****** |
|  | Full exclosure (-E-N)^c^ | -2.942 | 8.712 | 0.953 | ****** |
| *Uvaria caffra* | Open access (+E+N)^a^ | -2.539 | 7.464 | *.792 | ****** |
|  | Partial exclosure (-E+N)^b^ | -2.936 | 7.792 | 0.925 | ****** |
|  | Full exclosure (-E-N)^c^ | -2.677 | 7.926 | 0.922 | ****** |
| *Tricolysia junodii* | Open access (+E+N)^a^ | -4.152 | 8.466 | 0.741 | ***** |
|  | Partial exclosure (-E+N)^b^ | -3.847 | 7.883 | 0.613 | ***** |
|  | Full exclosure (-E-N)^c^ | -4.642 | 9.196 | 0.839 | ****** |
| *Dialium schlechteri* | Open access (+E+N)^a^ | -0.465 | 1.382 | 0.367 | ***** |
|  | Partial exclosure (-E+N)^b^ | -0.541 | 1.908 | 0.296 | ****** |
|  | Full exclosure (-E-N)^c^ | -0.352 | 1.505 | 0.151 | **-** |
| *Newtonia hildebrandtii* | Open access (+E+N)^a^ | -0.390 | 1.487 | 0.142 | **-** |
|  | Partial exclosure (-E+N)^b^ | -0.354 | 1.442 | 0.135 | **-** |
|  | Full exclosure (-E-N)^c^ | -0.338 | 1.397 | 0.087 | **-** |
| *Pteleopsis myrtifolia* | Open access (+E+N)­­ ^a^ | -0.689 | 2.387 | 0.357 | ***** |
|  | Partial exclosure (-E+N)^b^ | -0.519 | 1.981 | 0.352 | ***** |
|  | Full exclosure (-E-N)^c^ | -0.926 | 3.287 | 0.419 | ****** |

Regressions were performed for each of six tree species for each of the three exclosure treatments in the Sand Forest of Phinda Private Game Reserve, South Africa. A steep negative slope indicates an increasing population (i.e. a high abundance of seedlings and a low abundance of tall trees), whereas a negative slope close to zero indicates a disrupted distribution with many large trees and little regeneration.

^*^ P < 0.05; ^**^ P < 0.01; -, non significant (P > 0.05)

^a^ Unfenced area accessible for all herbivores

^b^ Area fenced to exclude elephant

^c^ Area fenced to exclude nyala and elephant, but accessible to smaller herbivores
